# MicroRNA-148a-3p suppresses cell proliferation and migration of esophageal carcinoma by targeting CEP55

**DOI:** 10.1186/s11658-021-00298-1

**Published:** 2021-12-24

**Authors:** Yong Lin, Yushan Chen, Rongqiang Shen, Dingzhu Chen, Yimin Lin

**Affiliations:** 1 Department of Cardiothoracic Surgery, Zhangzhou Affiliated Hospital of Fujian Medical University, No. 59 Shengli West Road, Zhangzhou, Fujian 363000 People’s Republic of China; 2 Department of Radiology, Zhangzhou Affiliated Hospital of Fujian Medical University, Zhangzhou, Fujian 363000 People’s Republic of China

**Keywords:** Esophageal carcinoma, microRNA-148a-3p, CEP55, Proliferation, Migration, Invasion, Apoptosis

## Abstract

This study evaluated microRNA-148a-3p in esophageal carcinoma cells. The prediction of bioinformatics analysis revealed that microRNA-148a-3p may target CEP55. qRT-PCR and western blot showed that CEP55 level in esophageal carcinoma cells and tissue was dramatically higher than that of normal cells and tissue, while microRNA-148a-3p was the opposite. Forced expression of microRNA-148a-3p restrained cell malignant behaviors of esophageal carcinoma, and repression of microRNA-148a-3p resulted in the converse results in terms of cell function. Dual-luciferase assay confirmed that microRNA-148a-3p targeted CEP55. CEP55 attenuated the suppressive effect of microRNA-148a-3p on proliferation and migration of esophageal carcinoma cells, demonstrating that microRNA-148a-3p regulated function of esophageal carcinoma cells via decreasing CEP55 level. Microscopy observation indicated that cell morphology was also affected by the microRNA-148a-3p/CEP55 axis. Furthermore, western blot analysis revealed that the PI3K/AKT signaling pathway could be suppressed by activating the microRNA-148a-3p/CEP55 axis. Finally, in vivo experiments confirmed the effects of microRNA-148a-3p on tumorigenesis. Thus, microRNA-148a-3p could act as a repressor in esophageal carcinoma via binding to CEP55.

**Supplementary Information:**

The online version contains supplementary material available at 10.1186/s11658-021-00298-1.

## Introduction

Esophageal carcinoma ranks as the sixth most prevalent malignancy in the world [1]. Despite recent advances in treatments, the five-year survival of esophageal carcinoma is still less than 20%, making it a fatal malignant tumor worldwide [1]. Given the complex factors that are responsible for esophageal carcinoma onset and progression, it is urgent to identify the main molecular mechanism of cancer progression [2–5], which will be beneficial for discovering new therapeutic targets for esophageal carcinoma.

MiRNAs usually suppress translation and stability of mRNAs and control genes related to cellular processes [6]. Therefore, miRNAs are involved in modulation of almost all signal transduction pathways in the cells, and their imbalance is key to development of cancers [7]. MiRNAs are usually produced through a multi-step process, starting from the nucleus and then entering the cytoplasm, consisting of three main processes: shearing, exporting and cutting [8].

Accumulating evidence has shown that miRNAs play a pivotal role in cancers. It is reported that microRNA-124-3p downregulates ITGB3 expression to suppress the malignant progress in gastric cancer [9]. Bo et al. [10] found that microRNA-30a-3p/5p directly binds to Wtn2 and Fzd2 to hinder the Wnt signaling pathway, thus repressing proliferation of esophageal carcinoma cells. Moreover, it was reported that microRNA-214 targeted PD-L1, by which malignant progression of diffuse large B-cell lymphoma can be attenuated [11]. Although some miRNAs have been revealed to play a modulatory role in esophageal carcinoma, the mechanism of miRNA involvement in the pathogenesis of esophageal carcinoma is still poorly understood.

A study regarding epithelial ovarian cancer (EOC) revealed that microRNA-148a-3p can act as a tumor repressor via targeting c-Met in EOC, and microRNA-148a-3p is decreased in EOC tissue, while a lower microRNA-148a-3p level is related to a higher overall survival rate [12]. MicroRNA-148a-3p is also found less expressed in gliomas, where it targets TGIF2 and inhibits the cellular process of glioma in vivo and in vitro [13]. In these studies, microRNA-148a-3p acts as a cancer repressor and negatively regulates growth of cancer cells. CEP55 is a newly discovered member of the centrosome-related protein family that is involved in modulating the cell cycle. CEP55 is highly expressed in certain cancers. For example, in hepatocellular carcinoma, high CEP55 expression is reported to be upregulated in liver cancer [14], and its dysregulation is involved in malignant progression of liver cancer cell [15]. Furthermore, there are similar studies in liver cancer [14] and thyroid cancer [16]. However, there are relatively few studies on the mechanism linking microRNA-148a-3p and CEP55 to metastasis of esophageal carcinoma.

In this study, it was found that CEP55 level was higher in esophageal cancer, while the microRNA-148a-3p level was lower, and these two molecules were inversely correlated with each other. Also, CEP55 was directly targeted by microRNA-148a-3p in esophageal carcinoma, which is congruous with studies in other cancers. In vitro, the protein expression of CEP55 was pronouncedly reduced/improved by overexpressing/silencing microRNA-148a-3p in ECa-109and OE19, and the cell functions could be affected by this regulation. In addition, the results were corroborated in vivo to some extent. Our study demonstrates that microRNA-148a-3p and CEP55 interact in esophageal carcinoma. Meanwhile, it suggests that microRNA-148a-3p and CEP55 can be potential biomarkers, laying the groundwork for further investigation of the mechanism of esophageal carcinoma.

## Materials and methods

### Bioinformatics analysis

Numerous studies have shown that microRNA-148a-3p, as a tumor repressor, is underexpressed in various cancers [12, 13]. To assess microRNA-148a-3p level in esophageal cancer, The Cancer Genome Atlas (TCGA) database was applied for data downloading, including esophageal carcinoma-related miRNA expression data (normal: 13 and cancer: 165). MicroRNA-148a-3p level in the normal and cancer groups was determined. Target genes of microRNA-148a-3p were identified through miRDB, mirDIP, TargetScan, miRTarBase, and starBase databases. Esophageal carcinoma-related mRNA expression data (normal: 11 and cancer: 160) were downloaded from TCGA and were subjected to the edgeR package for differential expression analysis between the cancer and normal groups. Markedly upregulated genes were overlapped with the predicted target genes for follow-up studies.

### Clinical cases

Tumor tissue and matched adjacent tissue were collected from 30 esophageal carcinoma patients in the Zhangzhou Affiliated Hospital of Fujian Medical University between June, 2020 and December, 2020. Samples were frozen immediately in liquid nitrogen following collection and then stored at − 80 °C for preparation. All patients gave informed consent, and samples were approved by the ethics committee of the Zhangzhou Affiliated Hospital of Fujian Medical University.

### Cell culture and microscopy

Human esophageal carcinoma cell lines ECa-109 (BNCC337687), TE-1 (BNCC100151), and EC9706 (BNCC339892), OE19 (BNCC338566) and the normal cell line Het-1A (BNCC337688) were accessed from BeNa Culture Collection (BNCC, Beijing, China). All cells were maintained in RPMI-1640 medium plus 10% fetal bovine serum (FBS), 100 U/ml streptomycin and 100 U/ml penicillin in an incubator at 37 °C with 5% CO_2_. All culture materials were accessed from Gibco. Cell morphology was observed under an inverted microscope (ECLIPSE Ts2R, Nikon, Japan).

### Cell transfection

The oligonucleotides for regulating microRNA-148a-3p expression and corresponding controls (miR-NC mimics and microRNA-148a-3p mimics; miR-NC inhibitor and microRNA-148a-3p inhibitor) were obtained from ABM (ABM, Canada). According to the manufacturer’s instructions, the oligonucleotides were transfected into ECa-109 and OE19 cells using Lipofectamine 2000 (Thermo Fisher Scientific), and the transfected cells were maintained in corresponding medium under routine conditions. Next, the transfected cells were plated or harvested.

The CEP55 overexpression and silencing vector (oe-CEP55 and si-CEP55) were generated by GenePharma (Suzhou, China). Lipofectamine RNAiMAX (Thermo Fisher Scientific, USA) was applied for transient transfection with ECa-109 and OE19 cells to establish overexpression and silencing constructs. All functional tests were performed after 48 h of transfection.

### RNA isolation and qRT-PCR

Following traditional methods, total RNA was isolated from cells or tissue with TRIzol reagent (Invitrogen), and the RNA quantity was measured using a UV-2401 spectrophotometer (Shimadzu, Japan). Quantities of 1 mg of miRNA and mRNA were inversely transcribed into cDNA using the PrimeScript RT reagent kit (TaKaRa) and TransScript One-Step gDNA Removal and cDNA Synthesis SuperMix kit (AT311-02, TransGen Biotech, Beijing), respectively. The miScript SYBR Green PCR Kit (Qiagen, Hilden, Germany) and SYBR Premix EX TaQ II kit (Takara, China) were employed to detect miRNA and mRNA expression, respectively. The primers designed by TaKaRa for amplification included CEP55, microRNA-148a-3p, β-actin and U6, as detailed in Table 1. β-actin and U6 were applied as internal references for CEP55 and microRNA-148a-3p, respectively. For each group at least three independent replicates were used. Relative expression was normalized by the 2^− ΔΔCt^ method.

**Table 1:** Primer sequences in qRT-PCR

Target gene	Primer (5′–3′)
microRNA-148a-3p	F: 5′-CGCTCAGTGCACTACAGAA-3′
	R: 5′-GTGCAGGGTCCGAGGT-3′
U6	F: 5′-GCTTCGGCAGCACATATACTAAAAT-3′
	R: 5′-CGCTTCACGAATTTGCGTGTCAT-3′
CEP55	F: 5′-TGAAGAGAAAGACGTATTGAAACAA-3′
	R: 5′-ACTGTGGCTCCAAACTGCTT-3′
β-actin	F: 5′-CATGTACGTTGCTATCCAGGC-3′
	R: 5′-CTCCTTAATGTCACGCACGAT-3′

### Western blot

RIPA buffer (R0010, Solarbio) and protease inhibitors were applied for cell lysis at 4 °C for 30 min. After determining the protein concentration, the cell extracts were separated on SDS-PAGE (P0012A, Beyotime Biotechnology, Shanghai, China). The proteins were transferred onto PVDF membrane (ISEQ00010, Millipore, Billerica, MA, USA) that was then sealed in 5% skim milk at room temperature for 1 h. Subsequently, the membrane was maintained with primary antibodies against CEP55, PI3K, P-PI3K, AKT, P-AKT and then with HRP-labeled secondary antibody goat anti-rabbit IgG H&L. β-actin was used as an internal reference. All antibodies were obtained from Cell Signaling Technology (USA). Finally, ECL reaction solution (WBKLS0100, Millipore) was applied for color development by immersing the membrane into it.

### CCK-8 assay

Cells (2 × 10^3^ cells/well) were inoculated into a 96-well plate supplemented with 100 µL of medium. CCK-8 (Dojindo Labs) was implemented for measurement of cell viability. Cells were maintained for 5 days, and viable cells were calculated by measuring the optical density (OD) at 450 nm.

### Cell apoptosis assay

The Annexin V-FITC/PI Detection Kit (Abcam) and flow cytometry were utilized to detect apoptosis. The transfected cells were digested and then resuspended in binding buffer, followed by staining with Annexin V-FITC and PI. Flow cytometry analysis was done based on FCS Express software to detect the cell apoptotic rate.

### Wound healing assay

Cells were cultured to confluence. A scratch was generated by a 200 μL pipette tip. Cells were rinsed with PBS for non-adherent cell removal, and remaining cells were maintained for 0 h and 24 h in routine conditions. The cell scratch area was analyzed using ImageJ.

### Transwell invasion assay

Matrigel-coated Transwell inserts (8 μmol/L, Corning Corporation) in a 24-well plate were recommended. The cell suspension (200 μL) was dropped into the upper chamber while 400 μL of medium (10% FBS) was dropped into the lower chamber. 48 h later, the non-invading cells were discarded. Meanwhile, the cells attached to the bottom surface of the insert were fixed with 4% paraformaldehyde, followed by staining with 0.01% crystal violet. Then, the number of invading cells was counted under a microscope.

### Dual-luciferase reporter gene assay

The wild-type (WT) 3ʹ-UTR fragment of CEP55 mRNA containing possible binding sites of microRNA-148a-3p along with the mutant one (MUT) was amplified by PCR, and was subcloned into the pmiRGLO (Promega, WI, USA) luciferase vectors to generate luciferase reporter plasmids CEP55-WT and CEP55-MUT. The reporter plasmids plus microRNA-148a-3p mimics or miR-NC mimics were co-transfected into ECa-109 cells using Lipofectamine 2000. 48 h later, cells were collected and the Dual-Luciferase Reporter Assay System (Promega) was used for luciferase activity determination.

### In vivo validation

Our animal experiments were approved by the Animal Experimentation Ethics Committee of Zhangzhou Affiliated Hospital of Fujian Medical University. 6 nude mice were randomly grouped into two groups, where one was the microRNA-148a-3p agomir group and another one was the agomir NC group. The ECa109 cells pretreated with microRNA-148a-3p agomir and agomir NC (GenePharma, China) were injected into the dorsal flanks of 5-week-old nude mice, and they were maintained in a pathogen-free, constant temperature and humidity environment. Tumor size was measured and recorded using calipers every 5 days for 5 weeks, in which the size was calculated as follows: size = (length × width^2^)/2. The tumors were obtained and weighed once, killing mice on the last day. The tumor tissues were prepared for qRT-PCR analysis.

### Statistical analysis

All data from three independent experiments were presented as mean ± SD and statistically analyzed using GraphPad Prism software. The measurement results were subjected to one-way ANOVA or Student’s *t*-test. *P* < 0.05 means statistically significant.

## Results

### MicroRNA-148a-3p is less expressed in esophageal carcinoma cells

**Fig. 1:**
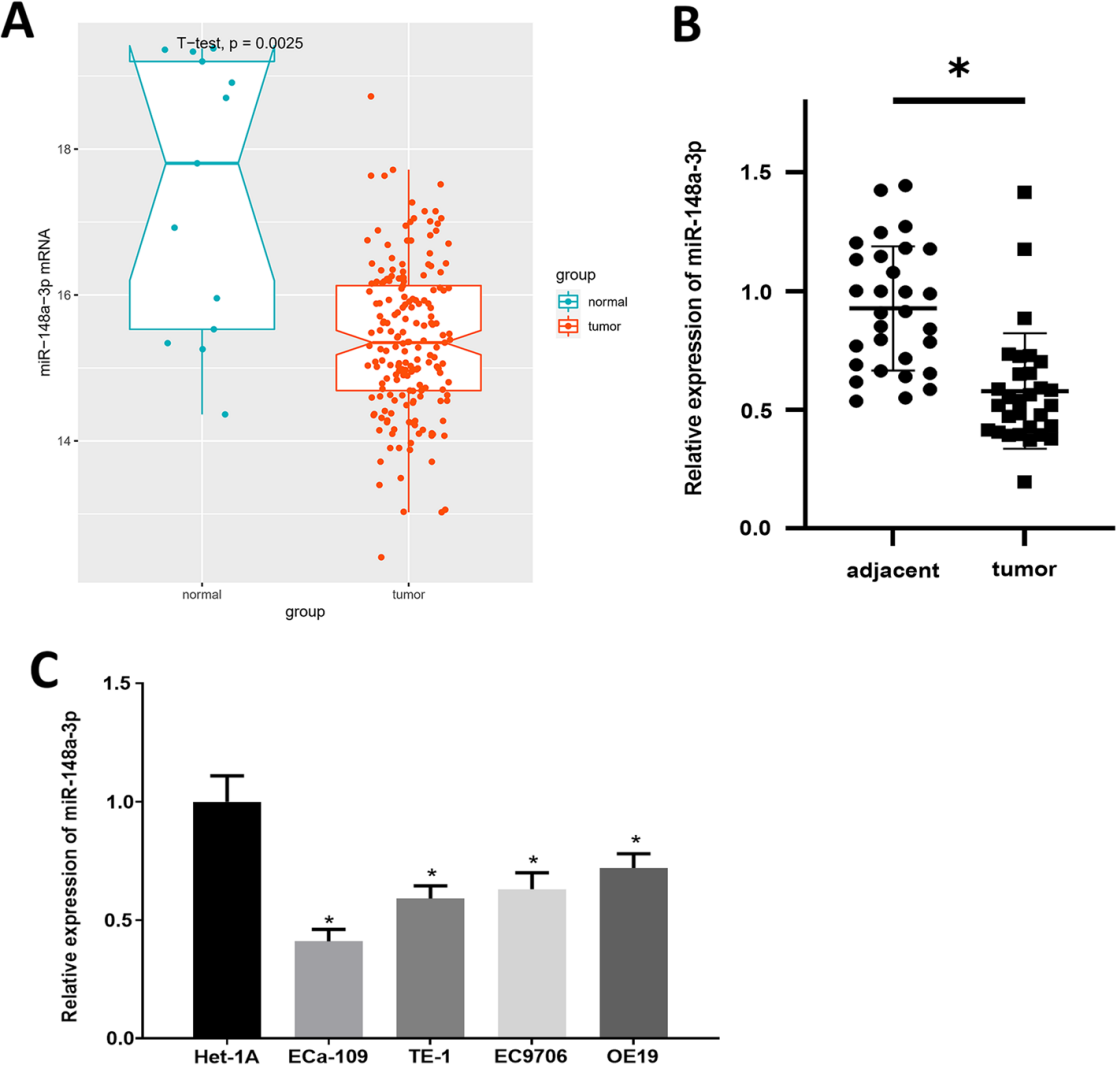
MicroRNA-148a-3p level is downregulated in esophageal carcinoma. **A** Box plot of microRNA-148a-3p expression of normal samples and tumor samples in TCGA-ESCA data set (row: sample type; column: expression level; left plot: normal samples; right plot: tumor samples); **B** MicroRNA-148a-3p level in tumor tissues and adjacent tissues. **C** MicroRNA-148a-3p level in ECa-109, TE-1, EC9706, OE19 and Het-1A cells was measured via qRT-PCR; *p < 0.05

Expression profiles of mature miRNAs (13 normal samples and 165 cancer samples) and mRNAs (11 normal samples and 160 cancer samples) of TCGA-ESCA along with their corresponding clinical data were from the TCGA database. Expression analysis of microRNA-148a-3p showed that microRNA-148a-3p was prominently less expressed in esophageal carcinoma tissue (Fig. 1A). To verify the expression status in our collected samples (n = 30), we analyzed microRNA-148a-3p level by qRT-PCR. The results indicated that microRNA-148a-3p was also downregulated in tumor tissue relative to normal tissue (Fig. 1B). In addition, survival analysis between high and low microRNA-148a-3p level was carried out based on the TCGA dataset (Additional file 1: Fig. S1A). Next, qRT-PCR was performed, and it revealed that microRNA-148a-3p level in esophageal carcinoma cells was lower than that in normal cells (Fig. 1C). Eventually, ECa-109 and OE19 were selected for subsequent functional assays.

### Modulation of microRNA-148a-3p expression affects cell proliferation, invasion, migration and promotes apoptosis of esophageal carcinoma

**Fig. 2:**
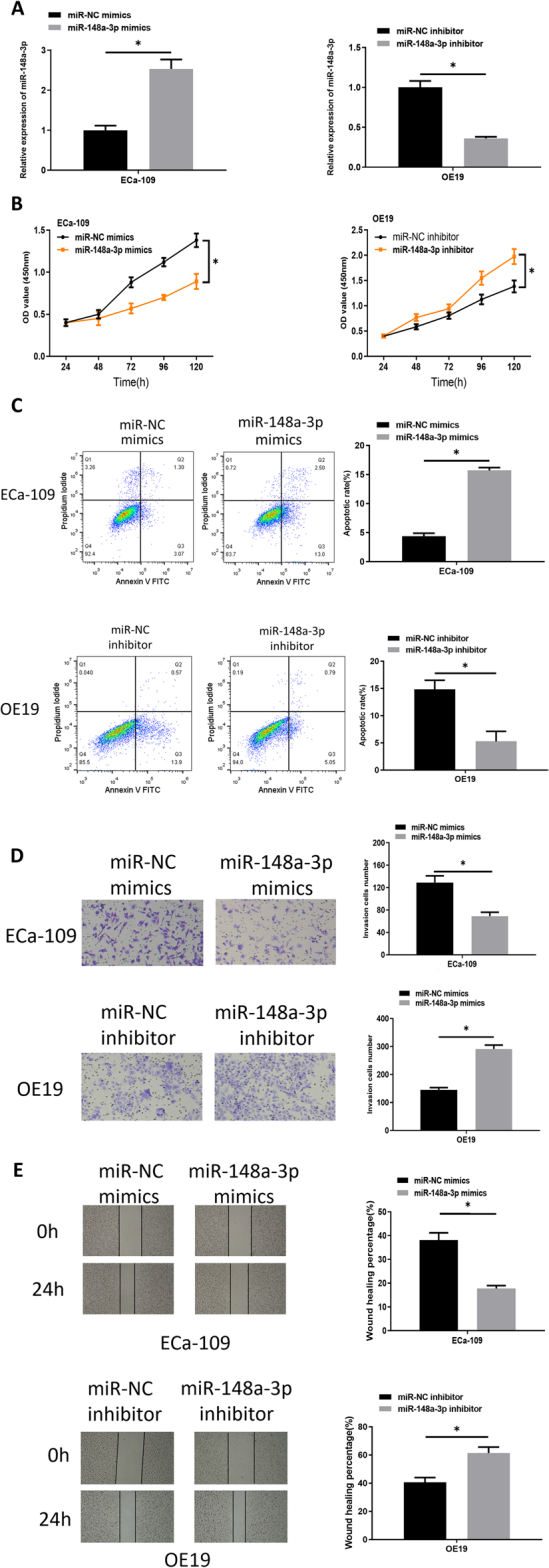
Regulation of microRNA-148a-3p expression inhibits cell proliferation, invasion, and migration, and promotes apoptosis of esophageal carcinoma. **A** MicroRNA-148a-3p was overexpressed in ECa-109 cells and downregulated in OE19 cells. **B** The difference in cell proliferation between the microRNA-148a-3p-regulated groups and the control groups was measured by CCK8. **C** The difference in cell apoptotic abilities of ECa-109 and OE19 cells before and after regulating microRNA-148a-3p was assayed via flow cytometry. **D** The difference in invasive ability of ECa-109 and OE19 cells between the microRNA-148a-3p-regulated groups and control groups was assessed through Transwell assay (100 ×). **E** The difference in migratory abilities of ECa-109 and OE19 cells between the microRNA-148a-3p-regulated groups and the control groups was measured via wound healing assay (40 ×); *p < 0.05

Since microRNA-148a-3p was prominently less expressed in esophageal carcinoma tissue and cell lines, it may play a tumor repressive role in esophageal carcinoma. To study the effects of microRNA-148a-3p on esophageal carcinoma cell function, microRNA-148a-3p overexpressed and downregulated cell lines were constructed based on ECa-109 and OE19, respectively. qRT-PCR was completed to assay microRNA-148a-3p overexpression and downregulation efficiency in ECa-109 and OE19 (Fig. 2A). Subsequent cell experiments illustrated that cell proliferation was markedly depressed by overexpression of microRNA-148a-3p while it was promoted by downregulation of microRNA-148a-3p (Fig. 2B). Also, it was noted that cell apoptosis was conspicuously increased after the microRNA-148a-3p level was raised, but the result was contrary with inhibition of microRNA-148a-3p (Fig. 2C). Moreover, Transwell and wound healing assay clarified that cell invasive and migratory properties were remarkably restrained with overexpressed microRNA-148a-3p, whereas introducing the microRNA-148a-3p inhibitor caused the opposite results (Fig. 2D, E). MicroRNA-148a-3p acted as a cancer suppresser in esophageal carcinoma and stimulated cell apoptosis.

### MicroRNA-148a-3p represses CEP55 level

**Fig. 3:**
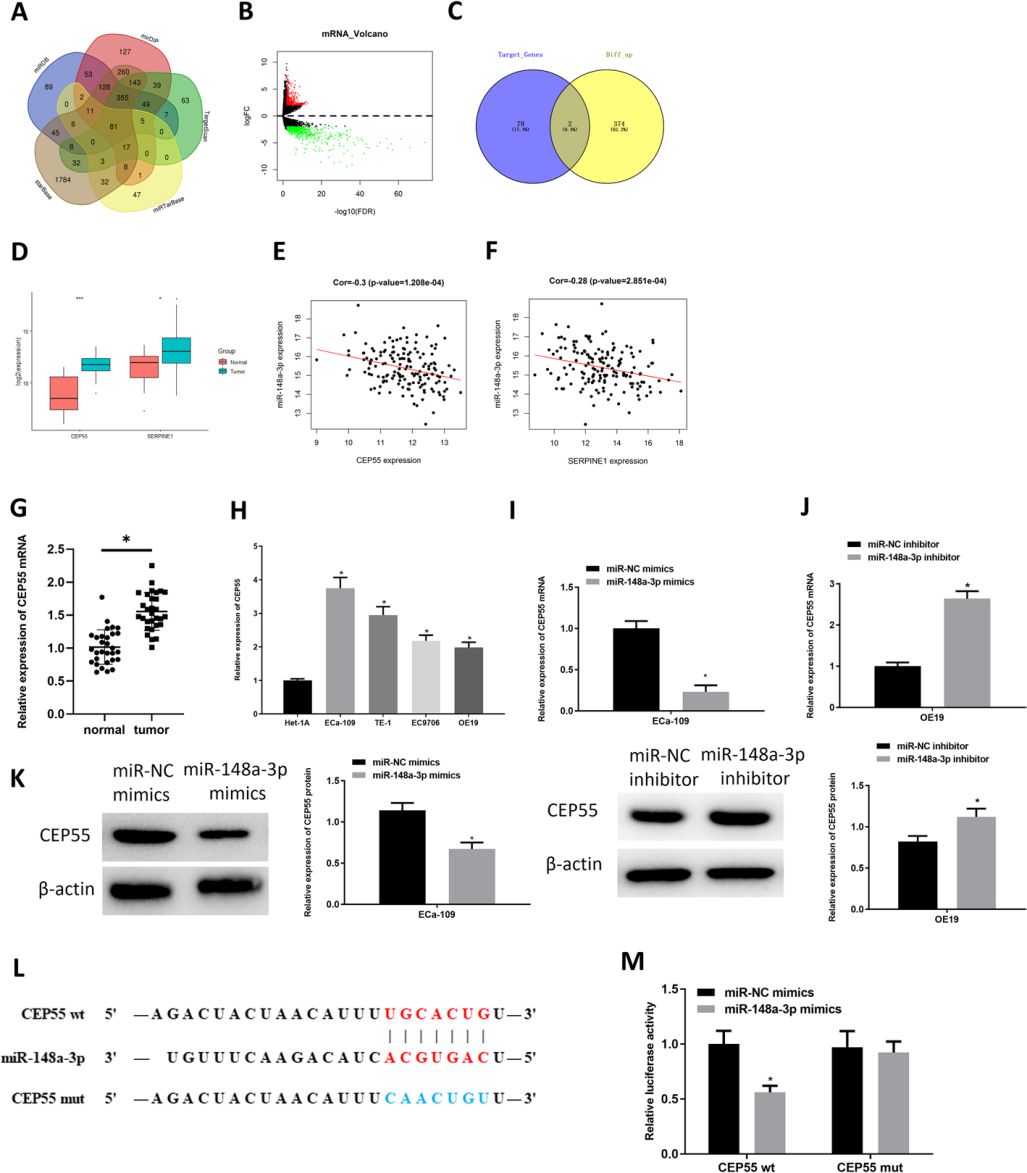
CEP55 is increased in esophageal carcinoma and is a target of microRNA-148a-3p. **A** Venn diagram of target genes of microRNA-148a-3p predicted by bioinformatics databases. **B** Volcano map of differentially expressed mRNAs (DEmRNAs) in TCGA-ESCA dataset. Red dots present significantly upregulated genes; green dots present significantly downregulated genes. **C** Venn diagram shows the intersection of the 81 overlapping mRNAs predicted by bioinformatics databases and the upregulated DEmRNAs in the TCGA-ESCA dataset. **D** Box plot of CEP55 and SERPINE1 levels in tumor and normal samples from TCGA-ESCA dataset. **E** Pearson correlation analysis for CEP55 and microRNA-148a-3p. **F** Pearson correlation analysis for SERPINE1 and microRNA-148a-3p. **G** CEP55 level in tumor tissues and adjacent tissues. **H** CEP55 level in ECa-109, TE-1, EC9706, OE19 and Het-1A cells was assessed through qRT-PCR. **I**, **J** Transfection efficiency of microRNA-148a-3p mimic and inhibitor. **K** CEP55 protein level of microRNA-148a-3p over-expressed and silenced cell lines. **L** Putative binding sites of microRNA-148a-3p on CEP55 3′UTR. **M** Dual-luciferase reporter gene assay verified binding relationship between microRNA-148a-3p and CEP55; *p < 0.05

Five highly recognizable and promising databases were used to identify targets of microRNA-148a-3p. As a result, 81 overlapping target genes were obtained and used for further analysis (Fig. 3A). Differential analysis for mRNA expression data of TCGA-ESCA (|logFC|> 2.0, padj < 0.01) was performed by edgeR, and 1002 differentially expressed genes (DEGs) were screened out, of which 376 were upregulated and 626 were downregulated (Fig. 3B). The up-regulated DEGs and the aforementioned 81 genes were intersected to obtain two key genes, CEP55 and SERPINE1 (Fig. 3C). Then, expression analysis and Pearson correlation analysis were carried out. The results illustrated that both CEP55 and SERPINE1 were highly expressed in esophageal carcinoma (Fig. 3D), and both were negatively correlated with microRNA-148a-3p (Fig. 3E, F). CEP55 was finally selected as the research object for better significant differential expression in esophageal carcinoma and higher correlation with microRNA-148a-3p. To determine the expression status of CEP55 in our collected samples, qRT-PCR was performed, and the results were consistent with our previous analysis (Fig. 3G). Moreover, survival and clinicopathological feature analyses were conducted for further research on CEP55 expression in esophageal carcinoma (Additional file 1: Fig. S1B). It was also verified via qRT-PCR that CEP55 was dramatically overexpressed in esophageal carcinoma cells (Fig. 3H).

Subsequently, we found that an increased microRNA-148a-3p level in ECa-109 cells led to the downregulation of CEP55 mRNA level, while repression of microRNA-148a -3p in OE19 resulted in upregulation of CEP55 mRNA expression (Fig. 3I, J). Also, results of western blot showed that overexpressed microRNA-148a-3p reduced the protein level of CEP55 as well, but inhibiting microRNA-148a-3p led to upregulation of CEP55 protein expression (Fig. 3K). Next, to further validate whether microRNA-148a-3p modulates CEP55 level, the TargetScan database was used to identify their binding sites, and it was found that microRNA-148a-3p and the 3ʹUTR of CEP55 mRNA had complementary regions (Fig. 3L). Then, dual-luciferase assay indicated that forced microRNA-148a-3p expression could significantly reduce luciferase activity of cells with CEP55-WT, with no significant effect on cells with CEP55-MUT (Fig. 3M). In summary, microRNA-148a-3p could down-regulate CEP55 expression.

### MicroRNA-148a-3p represses cell malignant phenotypes of esophageal carcinoma via modulating CEP55

**Fig. 4:**
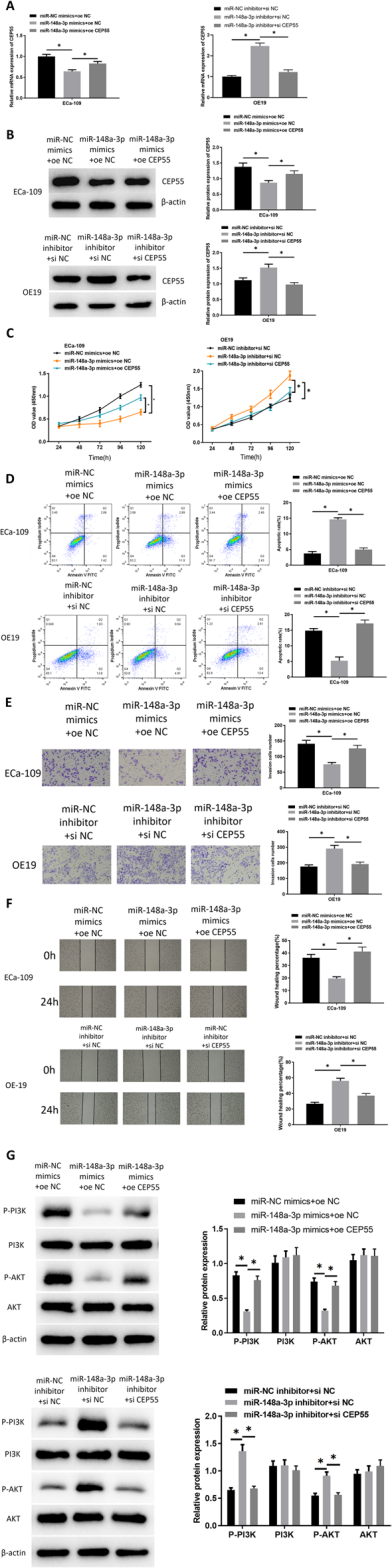
MicroRNA-148a-3p inhibits growth of esophageal carcinoma cells through inhibiting CEP55 expression. **A** CEP55 mRNA level in each group of cells measured through qRT-PCR. **B** Protein level of CEP55 in each group of cells was measured via western blot. **C** Cell proliferative ability of esophageal carcinoma in each transfection group was assessed by CCK8 assay. **D** Cell apoptotic ability of esophageal carcinoma in each transfection group was measured via flow cytometry. **E** Cell invasive ability of esophageal carcinoma in each transfection group was detected through Transwell assay (100 ×). **F** Cell migratory ability of esophageal carcinoma in each transfection group was assessed by wound healing assay (40 ×). **G** Western blot was applied to examine the PI3K/AKT related proteins *p < 0.05

The targeting impact of microRNA-148a-3p on CEP55 was initially assessed. Therefore, it was planned to construct co-transfected cell lines to evaluate their effects on cell functions. First, the expression of CEP55 mRNA in varying transfection groups was assayed by PCR, revealed that forced microRNA-148a-3p expression prominently decreased mRNA expression of CEP55, which was attenuated when CEP55 was overexpressed simultaneously. Inhibition of microRNA-148a-3p increased the mRNA level of CEP55, which could be attenuated by silencing CEP55 expression (Fig. 4A). Western blot was performed to detect the protein level of CEP55 in constructed cell lines, and the results revealed that protein expression of CEP55 was decreased remarkably with overexpressed microRNA-148a-3p, while it was increased significantly with inhibited microRNA-148a-3p. However, the expression of oe-CEP55 and si-CEP55 could recover the decreased and increased expression caused by the microRNA-148a-3p mimic and inhibition, respectively (Fig. 4B). The results of CCK8 assay for determination of cell proliferative ability are illustrated in Fig. 4C. In comparison to the control group, forced microRNA-148a-3p expression strongly hindered cell proliferation of esophageal carcinoma. In the microRNA-148a-3p mimics + oe-CEP55 group, compared with the microRNA-148a-3p mimics + oe-NC group, the proliferative ability of cells was conspicuously recovered. In contrast, for the microRNA-148a-3p inhibitor + si-NC group, cell proliferation was activated, which could be attenuated by the expression of si-CEP55. As for cell apoptosis, it was found through flow cytometry that overexpressed microRNA-148a-3p fostered cell apoptosis, while cell apoptosis was suppressed in the microRNA-148a-3p mimics + oe-CEP55 group. Downregulation of microRNA-148a-3p suppressed apoptosis, while this effect could be recovered by silencing CEP55 (Fig. 4D). Similarly, through Transwell and wound healing assays, enforced microRNA-148a-3p expression caused a notable reduction in cell migratory and invasive abilities, while this effect was offset by overexpressing CEP55. Downregulation of microRNA-148a-3p resulted in promoting cell migration and invasion significantly, which could be attenuated by silencing CEP55 simultaneously (Fig. 4E, F). Additionally, we observed cell morphologies under a microscope in the different transfection groups. The results showed that cells acquired a round shape after treatment with microRNA-148a-3p mimic, while overexpression of CEP55 could elongate the cells to recover their shape (Additional file 1: Fig. S1C), which related to cancer cell morphology. Also, a different study [17] showed that CEP55 level alteration in cancer cells affects the PI3K/AKT signaling pathway activity. Thus, we designed the following experiment to study whether the microRNA-148a-3p/CEP55 regulatory axis could affect the PI3K/AKT signaling pathway in esophageal carcinoma cells. The levels of proteins related to the PI3K/AKT signaling pathway in upregulated and downregulated microRNA-148a-3p groups were assayed via western blot (Fig. 4G). The results revealed that microRNA-148a-3p suppression could activate the PI3K/AKT signaling pathway, and vice versa. To sum up, it could be concluded that microRNA-148a-3p targeted CEP55 to restrain cell malignant behaviors of esophageal carcinoma via the PI3K/AKT signaling pathway, and the modulations of microRNA-148a-3p and CEP55 could affect cell morphology.

### In vivo functional experiments

**Fig. 5:**
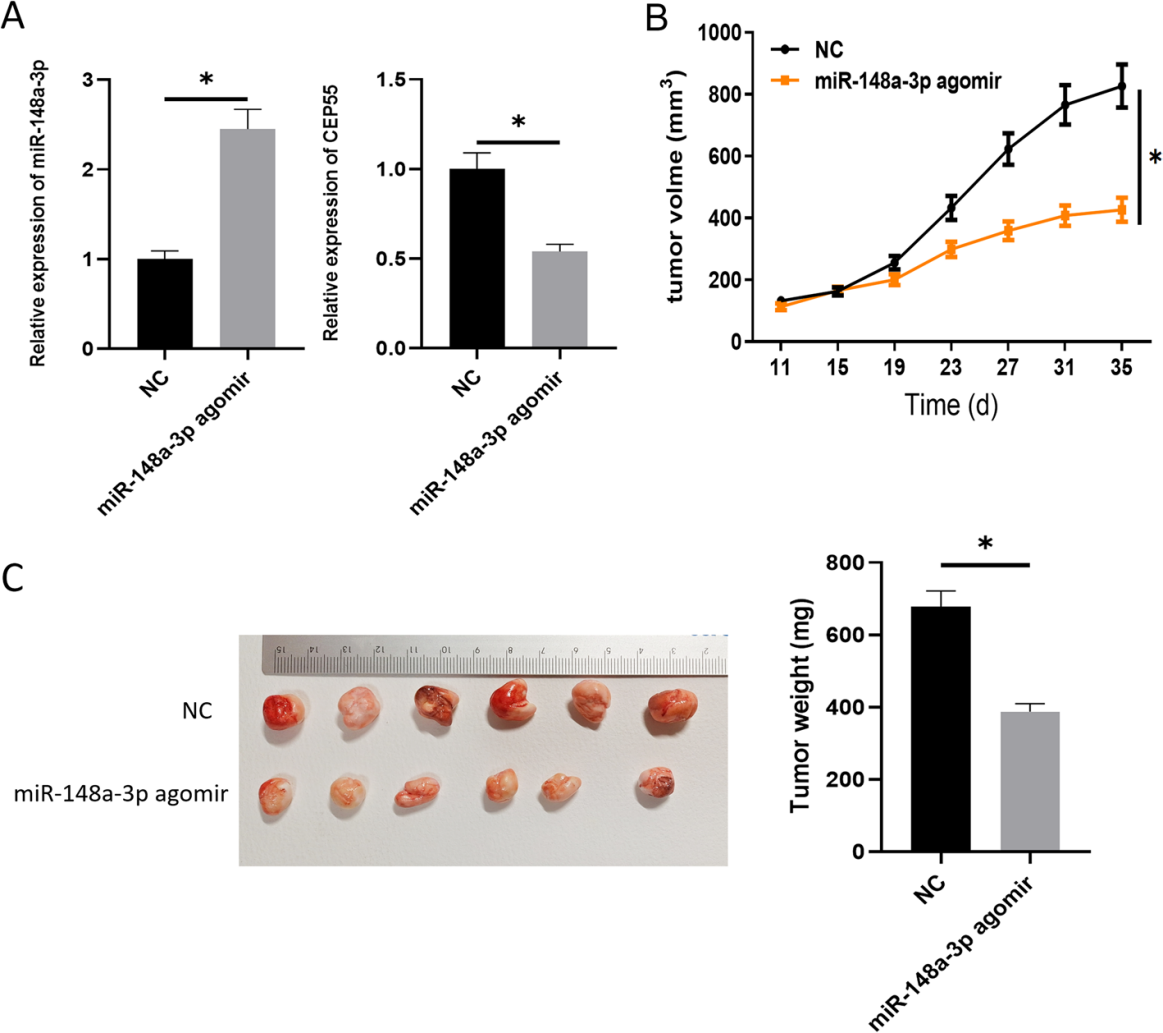
In vivo functional experiments. **A** MicroRNA-148a-3p and CEP55 levels in tumor tissues of mice model. **B** Tumor growth status in microRNA-148a-3p agomir group and control group. **C** Tumor size in microRNA-148a-3p agomir group and control group; *p < 0.05

To verify the role of microRNA-148a-3p in vivo, mice models were constructed using the treated cell lines. As expected, microRNA-148a-3p level was enhanced in the microRNA-148a-3p agomir group, while the expression of CEP55 was decreased (Fig. 5A). For tumor formation, the microRNA-148a-3p agomir group exhibited obviously smaller size and lower weight than the control group (Fig. 5B, C). Above all, as we expected, microRNA-148a-3p could repress CEP55 expression and tumor formation in vivo.

## Discussion

Esophageal carcinoma has inflicted rising morbidity in the western world in the past few decades [18]. MicroRNA-148a-3p and CEP55 play a pivotal role in many cancers, but their mechanisms of action underlying invasion and metastasis of esophageal carcinoma are poorly known. Therefore, this study looked deeper into the mechanism linking microRNA-148a-3p/CEP55 to esophageal carcinoma invasion and metastasis, hoping to discover a potential target for management of esophageal carcinoma.

MicroRNA-148a-3p was less expressed in esophageal carcinoma through bioinformatics analysis. This is congruous with the finding reported by Ma et al. [19] that microRNA-148a-3p is less expressed in oral cancer and regulates oral cancer progression via Akt signaling. MicroRNA-148a-3p is reported to decrease in gastric cancer (GC) cell lines with cisplatin (CDDP) resistance, and it enhances CDDP cytotoxicity in GC, briefly, which supports the potential of microRNA-148a-3p as a prognostic marker or therapeutic target to overcome CDDP resistance in GC [20]. Considering all the above, microRNA-148a-3p seems to be decreased in many tumors.

Next, microRNA-148a-3p expression was enforced, and overexpression of microRNA-148a-3p hindered malignant phenotypes of esophageal carcinoma cells. It is similar to the results from Wang’s research [12], which concluded that microRNA-148a-3p was correlated with overall survival of ovarian cancer, and overexpressed microRNA-148a-3p inhibited the invasive and proliferative abilities of EOC cell lines. As in their work, our results indicated significant anti-tumor effects of microRNA-148a-3p as well. However, there was no significant correlation between patients’ survival rates and microRNA-148a-3p expression.

CEP55 is a centrosome protein that participates in a variety of biological functions [21]. So far, it has been partly understood that CEP55 plays a pivotal role in a variety of cancers [14, 16, 22]. In fact, the effects of CEP55 on esophageal carcinoma have been studied on various levels [23–26]. For example, Jia Y’s work in 2018 demonstrated that CEP55 promotes the malignant progression of esophageal squamous cell carcinoma through the PI3K/AKT pathway [23], which is consistent with our results. However, the factors regulating CEP55 were not mentioned in the study, for which reason we designed our present work and revealed that microRNA-148a-3p could be one of the factors. In another paper, CEP55 is considered as a potential prognostic indicator of esophageal carcinoma [25]. However, our results did not support this conclusion by survival analysis. Possibly, the difference of applied sample databases may cause the different analysis results. In conclusion, though there are several studies concerning the impact of CEP55 on esophageal carcinoma, the views among them are not unanimous.

Our research suggested that microRNA-148a-3p regulated cell malignant phenotypes of esophageal carcinoma by targeting CEP55. Thus, the microRNA-148a-3p/CEP55 axis may be regarded as a possible target for management of esophageal carcinoma in the future. However, limitations still exist in our study. For one, the role of microRNA-148a-3p/CEP55 has not been fully validated in vivo. Therefore, we are planning to design experiments to comprehensively assess the role of microRNA-148a-3p/CEP55 based on in vivo experiments.

## Supplementary Information

**Additional file 1: Figure S1.** A: Survival analysis between high and low expression of microRNA-148a-3p; B: Survival analysis between high and low expression of CEP55; C: Cell morphology observation in the different transfection groups.

## Data Availability

The datasets used and/or analyzed during the current study are available from the corresponding author on reasonable request.

## Abbreviations

miRNAs: MicroRNAs
mRNAs: Messenger RNAs
EOC: Epithelial ovarian cancer
CEP55: Centrosome protein 55
BNCC: BeNa culture collection
FBS: Fetal bovine serum
oe-CEP55: Overexpression vector targeting CEP55
qRT-PCR: Real-time quantitative polymerase chain reaction
CCK-8: Cell counting kit-8
OD: Optical density
PI: Propidium iodide
WT: Wild-type
MUT: Mutant
ANOVA: Analysis of variance
DEGs: Differentially expressed genes
GC: Gastric cancer
