# Supplementary figures and images for: MicroRNA-148a-3p suppresses cell proliferation and migration of esophageal carcinoma by targeting CEP55
Source: Cell Mol Biol Lett. 2021 Dec 24;26:54. doi: 10.1186/s11658-021-00298-1 (PMC8903601; doi:10.1186/s11658-021-00298-1)

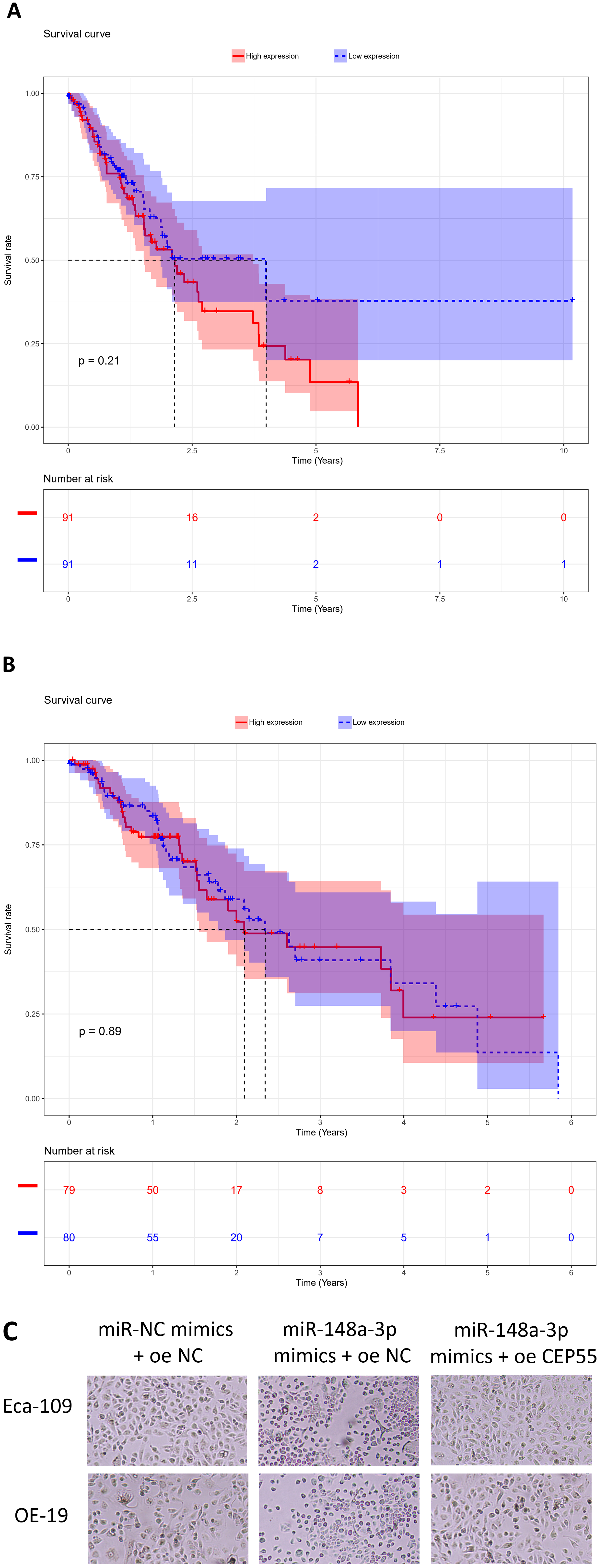

Supplement: Supplementary file 1 — Additional file 1: Figure S1. A: Survival analysis between high and low expression of microRNA-148a-3p; B: Survival analysis between high and low expression of CEP55; C: Cell morphology observation in the different transfection groups. [file 11658_2021_298_MOESM1_ESM.tif]
